# New Insights Into the Role of Aberrant Hippocampal Neurogenesis in Epilepsy

**DOI:** 10.3389/fneur.2021.727065

**Published:** 2021-12-15

**Authors:** Peng Chen, Fuchao Chen, Yue Wu, Benhong Zhou

**Affiliations:** ^1^Department of Pharmacy, Renmin Hospital of Wuhan University, Wuhan, China; ^2^Key Laboratory of Combinatorial Biosynthesis and Drug Discovery, Ministry of Education, Wuhan University School of Pharmaceutical Sciences, Wuhan, China; ^3^Sinopharm Dongfeng General Hospital, Hubei University of Medicine, Shiyan, China

**Keywords:** hippocampal neurogenesis, epilepsy, pathology, neural stem cells, therapy

## Abstract

Data accumulated over the past four decades have confirmed that adult hippocampal neurogenesis (HN) plays a key role in the wide spectrum of hippocampal pathology. Epilepsy is a disorder of the central nervous system characterized by spontaneous recurrent seizures. Although neurogenesis in persistent germinative zones is altered in the adult rodent models of epilepsy, the effects of seizure-induced neurogenesis in the epileptic brain, in terms of either a pathological or reparative role, are only beginning to be explored. In this review, we described the most recent advances in neurogenesis in epilepsy and outlooked future directions for neural stem cells (NSCs) and epilepsy-in-a-dish models. We proposed that it may help in refining the underlying molecular mechanisms of epilepsy and improving the therapies and precision medicine for patients with epilepsy.

## Introduction

Despite being assumed to be non-existent for decades, the occurrence and regulation of hippocampal neurogenesis (HN) in adult mammals has been widely accepted (1). Since various hormonal and environmental regulators are identified, this emerging form of adult brain plasticity is getting wide attention and has been studied (2). It is clear that these positive results further increase the interest in HN following the (re)confirmation that they existed in the adult human brain, which was paralleled by a drastic debate over the sequencing methods, disease model construction, and a possible reinterpretation of the functional role in the human brain (3).

Adult neurogenesis in the mammalian brain is a process by which functional neurons are generated from the division of neural stem cells (NSCs), which have a high capacity for long-term self-renewal while giving rise to the neurons and glia in the subventricular zone/olfactory bulb (SVZ/OB) system and hippocampal dentate gyrus (DG) from the embryo throughout the lifespan of animals (4). Conclusive evidence has confirmed that such a process in the adult subventricles and hippocampi was first documented in rodents, and then extensively explored in human postmortem brains (5). Specifically, granule neurons derived from adult hippocampal neurogenesis (AHN) in the DG are the first relay station in the information flow entering the hippocampus, and their firing rates are strongly regulated by different types of local interneurons, constituting an effective electrophysiological balance stabilizer. When disrupted by a variety of insults, such as brain injury that include stroke and status epilepticus (SE), there is hyperexcitability of hippocampal neurons and a variety of abnormal behaviors, such as memory deficits and decreased motor skills (6, 7).

The production of neurons decreases with age, possibly due to an alteration of the neurogenic niche in the SVZ, a developing environment, or the limited neurogenic capacity of NSCs in the brain (8). Over time, it may cause a progressive age-related depletion of the stem pool, although neuron production in the hippocampus can still be increased with the use of glucocorticoids and neurosteroids, or exposure of animals to environmental enrichment comprising physical activity (9). Several experimental studies on rodents have shown that AHN during aging is critically involved in learning, memory, and repair because the number and function of NSCs are reduced, resulting in fewer new neurons (10). Furthermore, the extent of cognition and certain types of brain repair are quantitatively linked to the time dependence of HN rates (11).

Over the years, multiple neurological diseases, such as epileptic seizures, ischemic stroke, Alzheimer's disease, and traumatic brain injury, have been demonstrated to be involved in the hippocampal neurogenic cascade in multiple ways (12). These changes have been primarily identified in epilepsy models and are referred to as aberrant HN, encompassing a wide variety of abnormal symptoms and alterations in the pathogenic cascade and size, spine numbers, morphology, function, and location of newborn granule neurons, which are fundamentally different from those under the stage of normal neurogenesis (13, 14). Here, we reviewed the recent findings of aberrant HN in epilepsy to improve our understanding of this field.

## Pathologies of AHN in Epilepsy

**Figure 1:**
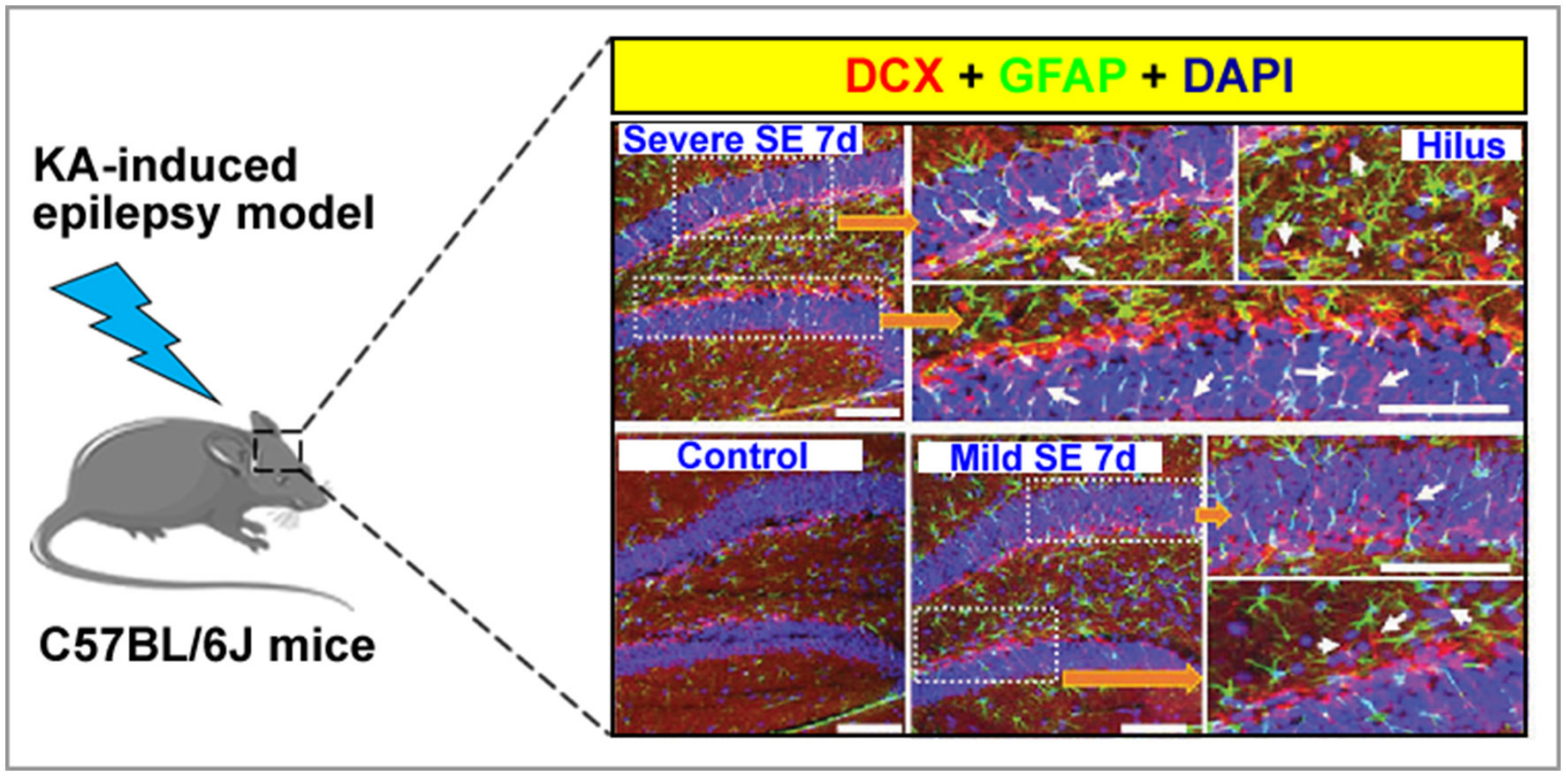
A graph showing normal and aberrant neurogenesis [stained with doublecortin, DCX (red)] in the dentate gyrus of a kainate-induced epilepsy mice model. Arrow indicated that newborn neuron.

Adult hippocampal neurogenesis in animals or humans is a multi-step process that includes migration, activation, proliferation, differentiation of local NSCs, and neuronal differentiation; thus, each stage may be more vulnerable to the dysregulation brought about by the pathological environment and external stimuli (15). In the case of seizures, multiple dysfunctional outcomes have been identified in rodent animal studies (Figure 1) (16). These changes mainly include an imbalance in quiescence and activation of adult NSCs, alterations in self-renewal rate of NSCs, decrease in the proliferative capacity of NSCs, neural progenitor cells, or neuroblasts, existence of aberrant integrations-hilar basal dendrites, hilar ectopic migration, mossy fiber sprouting in the DG-generated cells, and aberrant migration of newborn neurons into the dentates (17, 18). In particular, the development of an abnormal dendritic tree has been confirmed to be a significant feature of AHN because low synaptic connectivity prevents immature neurons from responding broadly to cortical activity, potentially contributing to an imbalance between excitation and inhibition (19). These abnormalities in the number and morphology of newborn neurons can result in the recruitment of newly generated neurons into functional hippocampal networks, creating recurrent excitatory circuits. Furthermore, excessive activation-coupled astrocytic differentiation of NSCs causes profound changes in the maturation-related phenotypes of neurons in the hippocampal DG and the function of hippocampal circuits (20). Thus, it can be hypothesized that the aberrant neurogenesis in the hippocampus is associated with recurrent seizures in patients with epilepsy. In addition, learning, memory, and psychiatric symptoms that include mood disorders, such as anxiety and depression, are affected by hippocampal neuroendocrine regulation and may become exacerbated. Moreover, AHN in epilepsy is likely to be commonly associated with brain dysfunction, leading to several behavioral comorbidities, such as anxiety and depression with learning and memory deficits (21, 22).

At present, among the common neurodegenerative diseases associated with AHN, one of the most studied is epilepsy and psychogenic non-epileptic seizures (23). Although the heterogeneities of the experimental animal models have been reported in the literature for epilepsy, it is universally acknowledged that epileptic seizures are often accompanied by an increasing rate of HN, followed by a steady decline (24). Furthermore, there is an abnormal development of morphological changes and functions in neurons generated in the animal hippocampus (5, 12). Remarkably, evidence from a recent study (25) of neurogenesis in the adult primate brain indicates that changes in neuronal long-lasting structures due to plasticity are a prominent feature of seizure-associated abnormal neurogenesis. In addition, the depletion of the NSC pool will be accelerated by the hyperactivation of NSCs caused by epilepsy, followed by a long-lasting decrease in NSC number at the later stages of AHN (26).

With the extensive research and understanding of stem cells, hyperactivation of NSCs has been studied in detail in recent years. Upon stimulation with inflammation and stress from convulsive seizures, quiescent NSCs (qNSCs, a type of slowly dividing cells) in the subgranular zone (SGZ) enter the cell cycle and become proliferative NSCs (27). Once activated after epilepsy, the activated NSCs (aNSCs) have the potential to principally divide asymmetrically to generate another NSC and amplify neural progenitors, which are representative of a pluripotent and highly proliferative state to maintain the NSC pool while expanding the progenitor pool (28). Asymmetric division coincides with cell fate determination, and newborn cells that generate aNSCs can further differentiate into reactive astrocytes through multiple rounds of asymmetric division, thereby contributing to increased astrogliosis (29). In general, AHN is regulated by both intrinsic and extrinsic cellular factors, and the complex regulation of AHN becomes clear when studying the fate of neural stem/progenitor cells (NSPCs) transplanted into ectopic locations in the brain (30). The well-documented neurogenic areas of the adult brain are the SGZ of the DG in the hippocampus, where new granule cells originate from NSPCs, and areas of the brain where astrocytes are neurogenic. These transplant studies indicate that the local environment or neurogenic niche in the epileptic brain is crucial for the development of NSCs and neurons (31). The hippocampal neurogenic niche is composed of a wide array of cell types, such as NSPCs, neuroblasts and their progeny, mature granule cells, astrocytes, GABAergic interneurons, microglia, macrophages, and endothelial cells connecting NSCs and their progeny to the vasculature (32, 33). Together, all these elements provide the hippocampus with a finely tuned microenvironment that is permissive for adult neurogenesis.

Understanding how physiological stimuli may affect neurogenesis through unique neural stem and progenitor cell populations may reveal mechanisms underlying phenotypic outcomes and provide novel therapeutic targets for disease (34). Interestingly, a recent study (35) revealed that significantly more dividing qNSCs and a corresponding increase in the number of surviving new neurons was observed in the hippocampi of kindled vs. sham-kindled rats. These findings are consistent with studies describing increased numbers of dividing qNSCs in the DG of rodents after electroconvulsive seizures or SE induced by chemo-convulsants kainic acid and pilocarpine (36). Thus, we speculated that qNSCs may be a novel target for the treatment of epilepsy and that aberrant neurogenesis could be a cellular mechanism of seizure development and maintenance. Future work employing complete neurogenesis ablation strategies (i.e., targeting resistant qNSCs) would be required to test whether aberrant neurogenesis (through the upregulation of qNSCs) is a cellular mechanism for epilepsy or physiological response to seizures. In the latter case, aberrantly connected young neurons could still provide a cellular substrate for seizure maintenance.

## Glial Function in Aberrant HN

Neurogenesis is a common physiological phenomenon that continues throughout life and represents the ability of brain cells to regenerate themselves. In most cases, adult neurogenesis in mammals is thought to be beneficial for maintaining physiological homeostasis in the brain and repairing neurological damage. For example, increasing adult HN is sufficient to reduce anxiety and depression-like behaviors in mice administered corticosterone (37). Moreover, enhancing optogenetic stimulation of adult-born neurons has been shown to specifically improve olfactory learning and long-term memory (38). Therefore, the role of neurogenesis in regulating emotional and cognitive functions has been recognized. A wide range of scholars have focused on this field, and much research has been conducted (39).

Recent studies have also suggested the beneficial roles of activated microglia in neurogenesis in the adult brain for providing structural, metabolic, and trophic roles for new neurons, phagocytic removal of dead cells, and modulation of the adaptive immune system in the central nervous system (40, 41). In addition, aberrant HN induced by acute seizures has been thought to be among the crucial players in the generation of spontaneous recurrent seizures and memory impairment (42). Whether microglial activity affects seizure-induced aberrant neurogenesis has only begun to be investigated in recent years.

Specifically, minocycline treatment led to a decreased activation of microglial cells, and the aberrant neurogenesis of the hippocampus after pilocarpine-induced acute seizures was attenuated, while this abnormal neurogenesis was promoted when the microglia were activated with lipopolysaccharide (43). More recently, it has been observed that the convulsive seizure-mediated aberrant neurogenesis was ameliorated by microglia in kainic acid (KA)-induced mice *via* the activation of toll-like receptor 9 (TLR9), a pattern recognition receptor of the innate immune system that recognizes microbial DNA and triggers inflammatory responses (44). In their study, the self-DNAs released from the damaged cells or neurons were sensed by TLR9 in microglia, which activated the NF-kB signaling pathway, causing aberrant seizure-induced neurogenesis. Another interesting study showed that microglia promoted seizure-induced aberrant neurogenesis through its P2Y12 receptor (P2Y12R) and increased seizure-induced immature neuronal projections between the DG and CA3 regions (45). Their results identified microglial P2Y12R as an important regulator of neurogenesis and suggested that targeting it may be a potential method for pro-epileptogenic processes (46). Taken together, these data suggest that microglia are involved in the regulation of the phenotype of acute epileptic seizures and play an important role in the proliferation, survival, and development of NSCs and neuritis during epileptic seizures (45).

It is still uncertain what triggers microglia, and a matter of debate whether microglial activation is beneficial or detrimental to neuronal death and neurogenesis following seizures (47). Therefore, there is no consensus on whether they are neuroprotective or neurotoxic in the human brain associated with epileptic seizures. To clarify the physiological and pathological roles of microglia in brain homeostasis and AHN in epilepsy, investigations on the interaction between the cells and their extracellular environment outside of the adult brain parenchyma are essential for researchers (39, 42). Recently, it has also been confirmed that there is a disruption in the structure and function of the blood-brain barrier in the progression of epilepsy, which suggests that cerebrovascular accidents may contribute to microglia-mediated neurogenesis in epilepsy (48). To prevent the development of epilepsy, it is crucial to discover the cellular and molecular mechanisms underlying the synaptic excitatory and inhibitory (E/I) imbalance of AHN during epileptogenesis (49). Furthermore, to prevent the development or progression of epilepsy, it is crucial to reveal the specific cellular and molecular mechanisms underlying synaptic E/I imbalance produced by AHN during epileptogenesis (50). Even if epilepsy has long been considered a synaptopathy and microglia are essential for the development of functional neural circuits, there have been no studies investigating the relationship between microglia and synapses in the AHN of epilepsy.

Astrocytes are also an important cell type in the neurogenic niche and provide a special environment for adult neurogenesis. Similar to microglia, astrocytes serve as important mediators that drive immune responses and promote inflammation. Inflammation is considered one of the greatest causes of neurogenesis, but some cytokines released may have neuroprotective functions. Interleukin (IL)-6 and IL-1β are the cytokines released from astrocytes and are proposed to act as protectors in the promotion of neuronal differentiation (51, 52). A previous study found that stem cell factor restores NPC proliferation in IL-6 knockout mice and that both hippocampus-dependent cognitive functions and the level of adult neurogenesis are gradually attenuated (53). Similar to the results of previous *in vivo* studies, there was also an improvement in astrocyte-specific IL-6 knockout mice (54). These results provide evidence that astrocytic IL-6, produced under physiological conditions, promotes neurogenesis and supports cognitive function. However, contrary to these findings, overexpression of astrocytic IL-6 decreased neurogenesis and alleviated hippocampus-dependent learning (55).

## Roles of microRNAs in Aberrant HN

The relief of AHN caused by epileptic seizures has become a hot topic of current research, and scholars have put forward many assumptions during this period. In recent years, miRNAs (small non-coding RNAs) have gained significant attention as accumulating evidence has shown that most miRNAs play a crucial role in regulating AHN, and are also deregulated in the cases of seizures or chronic epilepsy (56). Here, an overview of AHN-regulating miRNAs associated with epilepsy was presented based on the analyses performed using EpimiRBase developed by Mooney et al. (57). EpimiRBase is a searchable database (one of the largest manually curated target databases) indexing more than 2,000 miRNA sequences and annotations linked to epilepsy, which were developed to address the rapidly increasing need to track the progress of published literature on miRNAs related to epilepsy-induced aberrant AHN. The recently published EpimiRBase has updated miRNAs from more than 54 publications, and the results showed that 2,183 unique sequences (1,208 upregulated and 975 downregulated) have been found, which mainly consist of three species: humans, mice, and rats (58). Through an integrated analysis of these original miRNAs, they can be categorized into three classes based on their molecular functions: expression analysis, functional, and profiling-biofluid in the brain. Numerous matches have been provided by searchin EpimiRBase against the families of the upregulated and downregulated select miRNAs, which have also been implicated as crucial regulators of AHN, highlighting the crucial role of miRNAs in regulating AHN after SE (59).

The list of miRNAs that regulate AHN continues to grow, and AHN-focused miRNA-based therapy may be a promising strategy for epilepsy, although the complexity by which miRNAs regulate biological processes makes it challenging. Interestingly, several attempts to identify miRNA-based anti-epileptic therapies have been performed, although they have not primarily focused on AHN (56–58). Accumulating evidence suggests that the alterations in miRNA-34a expression may be involved in the pathogenesis of epilepsy (60). Two different rodent epilepsy studies have undertaken miR-34a silencing approaches to rescue seizure-induced apoptosis (61, 62). One study found that neuronal apoptosis in CA1 and CA3 was successfully in remission after antagomir-34a administration during SE. However, this was not assessed in the DG; thus, caution should be exercised when extrapolating these findings to AHN (61). Another study also silenced miR-34a using antagomirs, but no neuroprotective effects were observed after a 24-h post SE induction, indicating a potential time-dependent treatment window (62). From these data, we concluded that antagomir-34a administration has no beneficial effects on the duration and severity of seizures.

More compelling evidence for the role of miRNA-based regulation in epilepsy implications for therapy comes from several studies on developing possible targets for antagomir-134 anti-epileptogenic effects (63). As mentioned before, miR-134 is known to be involved in regulating synapse formation and dendritic spinogenesis; thus, it may be a key regulator of intrinsic excitability and susceptibility to seizures (64). A significantly greater proportion of pilocarpine-induced SE has been observed in developing rats after pretreatment of mice with antagomir-134 over 24 h before SE induction (65). In addition, in antagomir-treated mice that developed SE, seizure onset was delayed and the total seizure power was reduced. In another study, the results showed that the later occurrence of spontaneous seizures was induced by over 90% after depletion of miR-134 1 h after the onset of SE (66). Furthermore, the results indicated that miR-1 antagomir treatment significantly improved the neurological deficits and reduced CA3 pyramidal spine density, hippocampal astrogliosis, and neuronal cell loss, which are the pathological histological forms of temporal lobe epilepsy (TLE). Despite the above studies on miR-34a, the effects on AHN-related pathology were not measured.

## Organoids and Crispr/Cas9 to Study Genetic Epilepsy Syndromes

**Figure 2:**
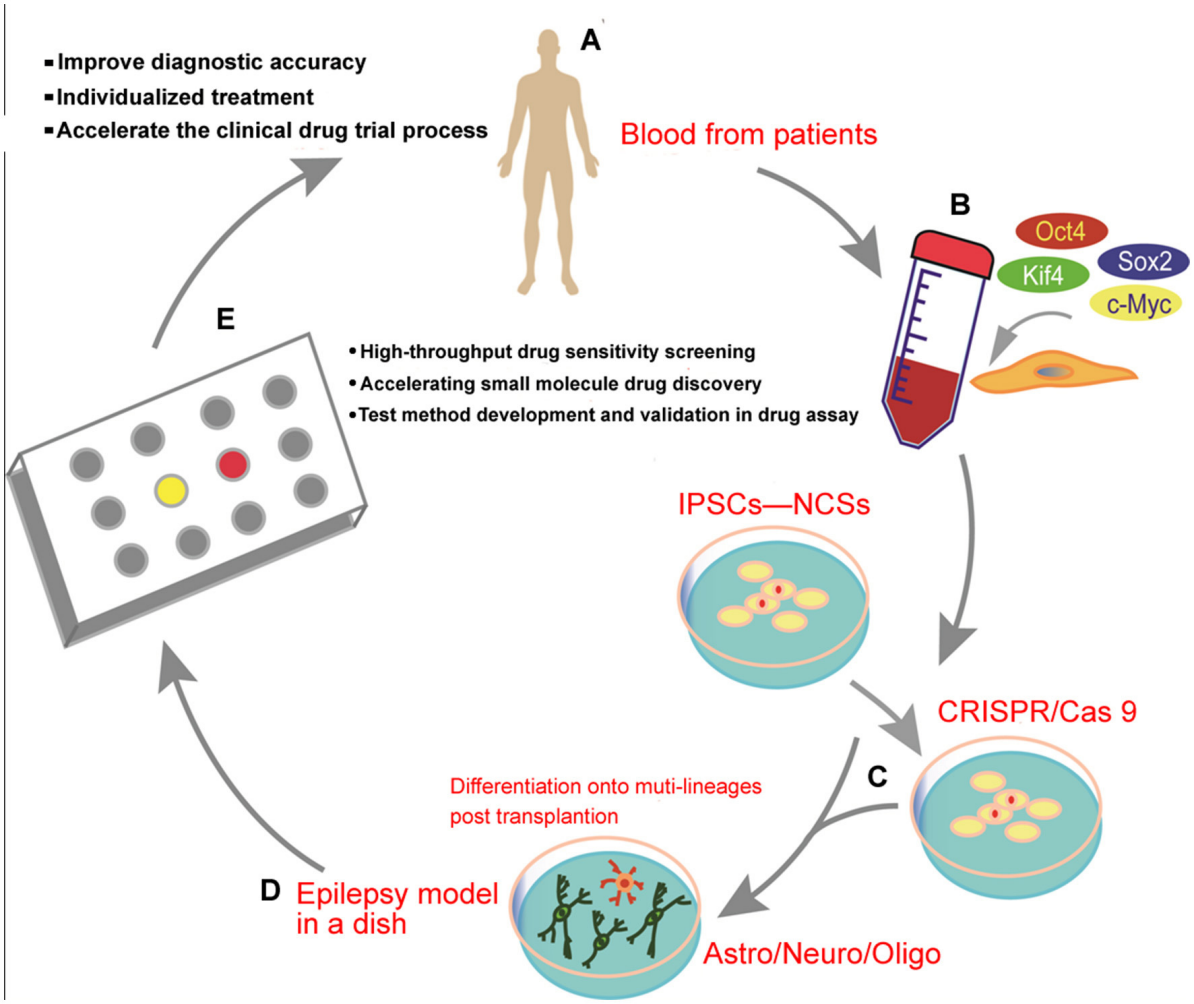
Schematic diagram illustrating the transformation of human-induced pluripotent stem cells (iPSCs) from the clinical trial in a dish to precision medicine for epilepsy: **(A)** clinical patient recruitment, **(B)** generation of human iPSCs from the blood of the patients, **(C)** gene targeting can be effectively achieved using the CRISPR/Cas9 editing system, **(D)** *in vitro* generation of human iPSC-derived brain organoids from a mixed population of neurons and glial cells, and **(E)** drug discovery, individualized therapy, or a clinical trial using the epilepsy-in-a-dish model.

A clear understanding of the early stages of epilepsy development is essential for a thorough investigation into new therapeutic strategies and target discovery and development. A large number of studies have shown that the combined application of rodent models *in vivo* and human-induced pluripotent stem cells (iPSC)-NPS models *in vitro* is a hot topic for future research on epilepsy (67, 68). Although great efforts have been devoted to elucidating many aspects of the environment of the epileptic brain, the understanding of epilepsy development remains elusive due to the significant difference between the human and rodent physiology models (69). The cell field has been transformed by technological advances in recent decades. The establishment and use of an epilepsy model in a dish have already been artificially realized (70). Human cells can be directly harvested for targeted editing of crop genomes using the CRISPR/Cas system, which is now entering a new era of personalized medicine. Moreover, it has been a hot topic in the medical field, where electrophysiological and histological changes in patients with epilepsy can be erased and studied without the need for human nerve tissue, which is available to patients with both surgical and autopsy specimens (71). In the future, we expect that it will be a motivation to expand the applications of the combination of *in vivo* rodent models and *in vitro* patient-derived iPSC models (Figure 2) to help us understand the different aspects of epilepsy and other neurodevelopmental disorders (72). Furthermore, we hope that more complex iPSC-derived epilepsy model systems can be developed and utilized in the screening of drug candidates, probing disease mechanisms, and advancing novel therapies.

## Conclusion

Epilepsy is a set of neurological disorders characterized by recurrent seizures and significant comorbidities. The role of NSCs in epilepsy is well-described, but the exact role of AHN in epileptogenesis remains to be elucidated. Moreover, there is comparatively little co-development of therapies and tests based on our current understanding of epilepsy. Given the rapid advances and the increased reliance on biotechnology, the iPSC-derived models hold strong promise for generating more relevant human physiological systems for drug testing, elucidating disease mechanisms, and developing new epilepsy therapies by bridging the gap between model systems and patients.

## Author Contributions

PC and BZ conceptualized and designed the study, analyzed, and interpreted data. PC wrote the manuscript. PC, FC, and WY designed the figures. BZ reviewed the manuscript.

## Funding

This study was supported by grants from the National Natural Science Foundation of China (31770381).

## Conflict of Interest

The authors declare that the research was conducted in the absence of any commercial or financial relationships that could be construed as a potential conflict of interest.

## Publisher's Note

All claims expressed in this article are solely those of the authors and do not necessarily represent those of their affiliated organizations, or those of the publisher, the editors and the reviewers. Any product that may be evaluated in this article, or claim that may be made by its manufacturer, is not guaranteed or endorsed by the publisher.
